# End-of-life sedation and gendered narrative registers: a longitudinal qualitative study of spouses bereaved by cancer

**DOI:** 10.1007/s00520-026-11224-6

**Published:** 2026-09-17

**Authors:** Yasmine Chemrouk, Livia Sani, Marthe Ducos, Pascal Gauthier, Marie-Frédérique Bacqué

**Affiliations:** 1 CHU Orléans, 14 Av. de l’Hôpital, 45100 Orléans, France; 2 University of Strasbourg, SULISOM UR 3071, 67000 Strasbourg, France; 3 Department of Health Science and Public Health, Faculty of Medicine and Surgery, Catholic University of the Sacred Heart, 00168 Rome, Italy; 4 Institut Bergonié, Bordeaux, France

**Keywords:** Cancer, Continuous deep sedation until death, Gendered narrative registers, Palliative care, Qualitative longitudinal study, Spousal bereavement

## Abstract

**Purpose:**

This study investigates gendered narrative registers in spousal grief following cancer, within this sample and interview context, in palliative care settings including continuous deep sedation until death (CDSUD). It examines (1) how men and women narrate and experience bereavement and (2) how their emotional, relational, and cognitive processes evolve across the first year of bereavement (3, 6, and 12 months post-loss).

**Methods:**

This longitudinal qualitative study is part of the AfterSedatio project. Twenty-two bereaved spouses (12 men and 10 women) were interviewed at 3 (T0), 6 (T1), and 12 months (T2) after the loss of their partner. Interviews were analyzed using ALCESTE lexical analysis, complemented by a codebook (coding reliability) thematic analysis.

**Results:**

Gendered patterns emerged across time. At T0, both men and women devoted substantial narrative space to the final days and the circumstances of the death, but in contrasting registers: women recounted these moments in sensorially detailed, emotionally embodied terms—bodily decline, acute care, and CDSUD decision-making—and framed the illness within the couple’s and the family’s biography, whereas men recounted the same period at greater emotional distance, in a factual register organised around clinical management, logistics, and prior family losses. At T1, the dominant classes for both genders were lexically weakly specified; within these, women’s accounts more often foregrounded emotional vulnerability and intrusive end-of-life memories and men’s routine disruption and relational absence. By T2, women tended to approach future planning cautiously and maintain strong emotional bonds, while men more often re-engaged in social life and personal projects. Across genders, the quality of palliative communication and clarity about sedation practices shaped grief integration. Women in this sample showed markedly higher financial vulnerability after bereavement.

**Conclusion:**

Within this sample and interview context, gender was associated with distinct narrative registers in how bereavement was narrated after cancer-related spousal loss, registers that were fluid rather than fixed. Emotional, relational, and pragmatic registers evolve, underscoring the need for individualized, gender-sensitive bereavement support. End-of-life communication, particularly around sedation, plays a critical role in later grief adjustment.

**Supplementary Information:**

The online version contains supplementary material available at https://doi.org/10.1007/s00520-026-11224-6.

## Introduction

Conjugal bereavement is a profoundly destabilizing life event that disrupts emotional, relational, and identity continuity, often resulting in psychological distress and social isolation [1, 2]. Beyond its emotional impact, widowhood also entails socioeconomic consequences, particularly for women, who show higher risks of financial decline and long-term instability [3, 4].

The trajectory of grief varies widely according to individual, relational, and contextual factors [5]. Gender constitutes one of these factors, influencing how people express and cope with grief: women tend to rely on emotional and relational processing, whereas men more often engage in pragmatic or restrained forms of expression [1, 6, 7]. These patterns reflect broader sociocultural norms [8, 9] and make gender a relevant analytic lens.

Cultural and religious frameworks further shape gendered grieving patterns, influencing acceptable emotional expression and coping practices [10].

Cancer is a particularly salient context for conjugal bereavement, as spouses are often deeply involved in caregiving throughout a prolonged illness trajectory. This involvement may generate anticipatory grief and heightened vulnerability but also potential for meaning reconstruction [11]. In advanced disease, caregiving typically coincides with palliative care, including practices such as palliative sedation delivered within ethical and clinical frameworks [12]. In France, continuous deep sedation until death (CDSUD) is legally framed by the 2016 Claeys–Leonetti law, which grants patients meeting specific end-of-life criteria the right to continuous and deep sedation maintained until death. The quality of end-of-life communication and decision-making can strongly shape the subsequent bereavement experience [13].

Despite increasing attention to gender differences, few studies have examined spousal bereavement in oncological and palliative contexts using qualitative longitudinal methods. Existing research largely relies on cross-sectional designs, limiting insight into how meanings and coping processes evolve over time [14].

This study, conducted within the French AfterSedatio project, addresses this gap by exploring (1) how men and women narrate and experience spousal bereavement in a palliative care setting and (2) how their emotional, relational, and cognitive processes evolve across the first year following the loss (3, 6, and 12 months post-bereavement).

This study used a critical realist epistemological framework [15], consistent with the coding reliability approach to thematic analysis adopted here and with the corpus-driven, lexicometric logic of ALCESTE.

## Materials and methods

### Participants

Between September 2022 and March 2025, fifty bereaved spouses were recruited from nine hospital sites across France, primarily palliative care units, in collaboration with medical and psychological teams. Eligible participants were adults (≥ 18 years), fluent in French, without cognitive impairment, and bereaved following a partner’s cancer-related death after initiation of palliative care. Physicians and psychologists identified potential participants, provided study information, and obtained written informed consent; the first two authors [details omitted to allow anonymized review] then contacted consenting individuals and conducted all interviews.

Participants were recruited consecutively at each of the nine participating sites: throughout the recruitment period, medical and psychological teams informed every bereaved spouse who met the eligibility criteria, and no further selection was applied. No target sample size was set in advance; recruitment continued throughout the study period, and the analytic sample comprises participants with a complete three-wave dataset (see participant flow). The adequacy of this sample was assessed against the criteria of information power [16]: the aim of the study is narrow, the sample is dense in specificity (bereaved spouses of patients who died after a cancer trajectory managed in palliative care), the analysis draws on established theory on gendered grief, and the interviews were long and detailed (mean duration 44–81 min across waves). The cross-case analytic strategy would ordinarily argue for a larger sample; this is partly offset by the longitudinal design, which yielded sixty-six interviews from twenty-two participants and therefore a substantially greater volume of material per case than a single-interview design.

Of the fifty enrolled participants, eleven (22%) discontinued participation for personal or health-related reasons. Of the thirty-nine spouses remaining in the longitudinal follow-up, twenty-two had completed all three interviews at 3 (T0), 6 (T1), and 12 months (T2) post-loss and had verbatim transcripts available at the point at which the corpus was closed for analysis; the remaining seventeen had either not yet reached the twelve-month interview or did not yet have complete transcripts, transcription having been performed manually by the two interviewers. The present analysis therefore focuses on these twenty-two participants. Inclusion thus depended on recruitment date and transcription progress, not on any participant characteristic; among the baseline characteristics available, the excluded participants were comparable to those included in age, gender distribution, diagnosis, and type of end-of-life sedation received. Analysis was restricted to complete cases because the study examines the evolution of narrative registers across waves and because the lexical classification is performed separately for each gender-by-wave sub-corpus; retaining different speakers across waves would have confounded temporal change with changes in the corpus composition.

The final sample consisted of 12 men and 10 women aged 31–75 years. Most participants had children (men: 83.3% and women: 100%), and a majority were living alone at the time of the study (men: 75% and women: 60%). Solid tumors were the main cause of death (men: 83.3% and women: 70%). Women reported a greater economic impact of bereavement than men: 50% experienced a ≥ 50% reduction in income and 30% a ≤ 25% reduction, whereas 66.7% of men reported no financial consequences. End-of-life sedation practices were distributed across both genders: CDSUD was reported by 33.3% of men and 50% of women, intermittent/light sedation by 33.3% of men and 20% of women, and no sedation by 33.3% of men and 30% of women. Women generally took part in longer interviews than men. Detailed sociodemographic and clinical characteristics are presented in Table 1.

**Table 1:** Sociodemographic data of participants and deceased spouses by gender

	Men (*n* = 12)	Women (*n* = 10)
Age (mean (SD, min–max))		
Participants	62.17 (10.22, 40–75)	56.00 (14.64, 31–73)
Deceased	62.1 (10.95, 46–74)	60 (11.04, 40–73)
Children (*n* (%))		
Yes	10 (83.3)	10 (100)
No	2 (16.7)	0 (0)
Living alone (*n* (%))		
Alone	9 (75)	6 (60)
With children	3 (25)	4 (40)
Type of cancer (*n* (%))		
Blood	1 (8.3)	1 (10)
Solid	10 (83.3)	7 (70)
Brain	0 (0)	1 (10)
Other	1 (8.3)	1 (10)
Educational level (*n* (%))		
High school diploma	1 (8.3)	1 (10)
Professional diploma	1 (8.3)	1 (10)
Bachelor’s degree	3 (25)	3 (30)
Master’s degree	5 (41.7)	5 (50)
Secondary vocational diploma	2 (16.7)	0 (0)
Economic impact after spouse’s death (*n* (%))		
≥ 50% reduction in income	2 (16.7)	5 (50)
≤ 25% reduction in income	2 (16.7)	3 (30)
No reported financial impact	8 (66.7)	2 (20)
Sedation (*n* (%))		
*Kind of sedation*		
CDSUD	4 (33.3)	5 (50)
Intermittent/light sedation	4 (33.3)	2 (20)
None	4 (33.3)	3 (30)
*Timing*		
< 7 days	6 (50)	3 (30)
< 1 day	2 (16.7)	4 (40)
Interview length in minutes (mean (SD))		
T0	65.1 (23.9)	81.3 (48.6)
T1	46.0 (14.5)	56.3 (30.1)
T2	43.6 (17.4)	52.6 (25.3)

*Other—e.g., sarcomas and melanomas

### Procedure

Between September 2022 and March 2025, data were collected through semistructured interviews conducted online by two authors [details omitted to allow anonymized review] using BigBlueButton (BBB), a secure videoconferencing platform hosted and ethically approved by the University of [details omitted to allow anonymized review]. Each interview was audio-recorded and transcribed verbatim. Participants took part in one interview at each of the three scheduled time points (T0, T1, and T2); beyond these three planned waves, no additional or repeat interviews were conducted, and no field notes were taken.

Verbatim quotes included in this manuscript were translated from French to English for publication by two of the authors [details omitted to allow anonymized review], one of whom is bilingual. All coding and analyses, including the ALCESTE lexical analysis, were conducted on the original French corpus; translation was applied only to the illustrative quotations selected for the English-language manuscript.

The interviewers, both PhD-level clinical psychologists trained in psycho-oncology and bereavement support, sought to minimize the potential impact of their clinical roles by disclosing their professional background, avoiding leading questions, and engaging in regular reflexive debriefings.

Interviews were conducted with particular attention to the participants’ emotional sensitivity. To prevent retraumatization, we did not return transcripts to participants for feedback. Table 2 summarizes the interview guide.

**Table 2:** Interview guide categories and example questions, by time point (T0, T1, and T2)

T0 (3 months post-loss)	
Categories	Examples of questions
The deceased	*Could you tell me about the deceased? What was your relationship?*
The illness	*Could you tell me the story of your spouse’s illness?*
Place of the pandemic*	*Could you tell me how the pandemic has impacted your relationship with your spouse?*
The death	*How did the death occur? [includes sub-questions on sedation, e.g. “Did your spouse wake up after the sedation was put in place?”]*
Your grief (reactions and experience)	*How did you or are you experiencing your grief?*
T1 (6 months post-loss)	
Categories	Examples of questions
The deceased	*Could you tell me about the deceased? [focus shifts to ongoing thoughts, need to talk about him/her, belongings kept]*
The illness	*Do you think about your spouse’s illness?*
The death	*Do you think about the time of your spouse’s death?*
Your grief (reactions and experience)	*How did you or do you experience your grief?*
T2 (12 months post-loss)	
The deceased	*Could you tell me about the deceased? [same focus as T1]*
The illness	*Do you think about your spouse’s illness?*
The death	*Do you think about the time of your spouse’s death? [new closing item: “It will soon be a year since you lost your spouse. Do you think a ceremony would be good for you and your loved ones?”]*
Your grief (reactions and experience)	*How did you or are you experiencing your grief?*
The deceased	*Could you tell me about the deceased? [same focus as T1]*

*Asked only of participants whose spouse's illness or death occurred during the COVID-19 pandemic period

The interview guide evolved across waves. At T0 (3 months post-loss), it addressed the circumstances of the illness and of the death in detail, in addition to biographical and grief-related questions. At T1 and T2 (6 and 12 months), the guide shifted toward participants’ ongoing thoughts, intrusive memories, attachment to the deceased’s belongings, and grief processing, with a closing question about a commemorative ceremony added at T2. Table 2 below now reflects the full guide for each wave.

### Data analysis

Analyses were performed with ALCESTE 2018 (*Analyse Lexicale par Contexte d’un Ensemble de Segments de Texte*), a software program designed for the quantitative exploration of textual corpora [17]. Max Reinert initially developed ALCESTE in 1979 at the Jean-Paul Benzécri Laboratory (CNRS, Paris), where it segments text into Units of Context (UCs) that may consist of one or more sentences, determined by punctuation and lexical density. It then applies chi-square statistics to identify co-occurrence patterns, generating lexical classes that are ordered by degree of association and grouped hierarchically. The two interviewers produced transcripts manually and verbatim; interviewer turns were removed, and each interview was preceded by a starred variable line coding participant identifier, gender, and wave. Lemmatization was applied using the standard French dictionary supplied with the software, with no manual stemming and no additional stop-word list; forms occurring fewer than twice in a sub-corpus were excluded from the analysis. Six separate classifications were performed, one for each gender-by-wave sub-corpus. Each sub-corpus was submitted to the double descending hierarchical classification procedure, in which two classifications are performed on context units of two different lengths, both determined automatically by the software, each cut at a maximum of eight terminal classes; only classes proving stable across the two classifications were retained, the minimum number of ECUs required to retain a class being set automatically. The solution’s stability across the two classifications therefore determined the number of classes rather than a fixed a priori value. Supplementary Table S1 reports, for each run, the number of interviews, the size of the sub-corpus, and the percentage of elementary context units (ECUs) retained in the classification; retention ranged from 59 to 84%, falling below the conventional threshold of 70% for women at T1 (59%) and men at T0 (67%). Two classes were dominant in size but weakly specified lexically: men at T1, class 2 (maximum *χ*^2^ = 8) and women at T1, class 2 (maximum *χ*^2^ = 31). Both are best understood as residual categories, grouping the material not captured by the smaller and lexically distinctive classes of their run, rather than as coherent lexical worlds; the descriptions below are correspondingly provisional, and no comparative claim rests on them.

For each class, Tables 3 and 4 report class size as a percentage of classified ECUs, along with the characteristic reduced forms and their *χ*^2^ values. For each wave and gender, we additionally examined individual-level *χ*^2^ associations to identify participants whose narratives were most strongly associated with a lexical class atypical for their gender, thereby providing a basis for the contrasting-case analysis reported in the “Discussion” section.

**Table 3:** Lexical classes identified in women’s interviews at each wave (Alceste; descending hierarchical classification). Characteristic forms are reduced forms, listed by decreasing *χ*^2^; English glosses in brackets

Wave	Retention	Class	Label	% Classified ECUs	Characteristic forms (*χ*^2^)	Illustrative excerpts
T0	83%	1	**Couple and family history**	62.78	vie [life] 61; an [year] 55; vivre [to live] 55; enfant [child] 52; beau [beautiful] 35; mère [mother] 34; monde [world] 34; grand [great] 33; père [father] 26; famille [family] 26	“It’s not fair, Mom. I didn’t get to spend much time with my dad.” It’s hard to hear things like that. (QC) She was very close to her father. He was her dad. […] My son is very, very close to me. He sticks to me like glue. And she was glued to me. (DN)
		2	**Acute care and the final hours**	23.68	infirmier [nurse] 131; nuit [night] 119; dormir [to sleep] 107; matin [morning] 104; chambre [room] 98; soin [care] 96; médecin [doctor] 91; réveiller [to wake] 74; lit [bed] 65; hôpital [hospital] 60	At 11 p.m., I called the nurses because he was having difficulty breathing. In fact, I had already mentioned it in the afternoon. (MC) They withdrew fluid from him, I remember that, but how much, I couldn’t tell you. (DN)
		3	**Illness progression and treatments**	13.54	cancer 279; opérer [to operate] 192; traitement [treatment] 182; rendez-vous [appointment] 149; douleur [pain] 142; maladie [illness] 115; tumeur [tumour] 109; examen [test] 107; résultat [result] 107; scanner [CT scan] 95	With metastases in the peritoneum, resulting in peritoneal carcinomatosis, so they didn’t do anything else; they just closed it up. (CT) So we’re following this protocol, which isn’t too bad, without saying it’s good, and so the doctor is suggesting we sign up for another cycle. (MN)
T1	59%	1	**Everyday life with the children and the deceased’s belongings**^**†**^	23.40	aller [to go] 60; manger [to eat] 52; vacance [holiday] 49; fille [daughter] 45; maison [house] 43; revoir [to see again] 41; fils [son] 37; meuble [furniture] 33; acheter [to buy] 31; garder [to keep] 27	We have our little ritual when we go to see him at the cemetery: we each bring a rose, take five minutes alone, and then talk to him about whatever we want to say. (QC) We’re going to share the goal of the game, which is to share and help other children, and he got angry at me. Yes, I don’t want to; I want to keep everything. It’s Daddy’s. (MN)
		2	**Making sense of the loss: unanswered questions and emotional experience**^**†**^	76.60	pouvoir [to be able] 31; question 18; comprendre [to understand] 16; rendre compte [to realise] 15; trouver [to find] 15; normal 11; savoir [to know] 10; maladie [illness] 9; émotion 8; situation 7	You have to accept it. I know that it’s day-to-day anyway. (OS) There are so many questions, and it’s not easy to let go of them all and let what needs to be done happen. (MN)
T2	82%	1	**Evaluation of care and hospital services**	19.83	soin [care] 317; médecin [doctor] 142; service [ward] 101; hôpital 66; rendez-vous 62; malade [patient] 59; hospitaliser [to hospitalise] 55; urgence [emergency] 49; formidable [wonderful] 45; dégrader [to deteriorate] 35	In terms of palliative care, I was fine, everything was perfect. (QC) The emergency room, resuscitation, and the intensive care unit were violent, he said. It’s cowboys in the emergency room. It was very, very violent. The palliative care unit was gentle. (DN)
		2	**Life reorganization and the question of the future**	80.17	envie [desire] 21; vie [life] 20; faire [to do] 17; chose [thing] 15; enfant [child] 14; année [year] 14; deuil [grief] 10; père [father] 10; penser [to think] 9; aimer [to love] 8	I feel ready to start my life over and meet someone new. That too is the same. I didn’t think I would be able to do it. (ND) Basically, he told me—he told me he doesn’t want to get attached to them, because he’s nothing to them and will never be anything to them. I told him: today you’re nothing, true, on paper you’re nothing. But in practice, you’re not nothing—because you’re part of my children’s daily life. So in practice, you do have a status. (MN)

^†^Two narrative subthemes had previously been reported separately for this class; they are presented here within the single class from which they derive

Retention = percentage of elementary context units (ECUs) classified by the descending hierarchical classification for that wave; unclassified units are excluded from class-level percentages, which sum to 100% of classified ECUs within each wave. For each class, the ten forms with the highest *χ*^2^ are reported; where several forms share the same *χ*^2^ value, those listed are the first returned by the software

**Table 4:** Lexical classes identified in men’s interviews at each wave (same structure as Table 3)

Wave	Retention	Class	Label	% Classified ECUs	Characteristic forms (*χ*^2^)	Illustrative excerpts
T0	67%	1	**Illness history and its clinical management**	25.63	soin [care] 197; traitement [treatment] 108; médecin [doctor] 70; cancer 58; douleur [pain] 53; hôpital 47; tumeur [tumour] 41; foie [liver] 38; opérer [to operate] 35; séance [session] 32	She needed a few rounds of chemotherapy before she could have the operation, so the four rounds of chemotherapy she had had worked well enough for her to be able to have the operation. (UH) But it’s impossible to measure, but I think I’ll never experience that pain. I think it was hell for her. (QN)
		2	**The last days and nights**	52.48	dire [to say] 41; nuit [night] 39; matin [morning] 32; heure [hour] 31; faire [to do] 30; dormir [to sleep] 28; demander [to ask] 27; dernier [last] 23; seul [alone] 20; infirmier [nurse] 16	She had been fighting for quite a while, and you see, it was 8 p.m., and she passed away at 10:30 p.m. (KE) That weighs on me a little, but the nurses and physicians told me, “Don’t have any regrets.” But I was there, I was there the whole time during the three days that I needed to be there. (UH)
		3	**Couple history and previous family losses**	21.89	an [year] 114; mère [mother] 112; père [father] 91; parent 61; ensemble [together] 61; marier [to marry] 54; époux [spouse] 48; habiter [to live in] 47; cérémonie [ceremony] 47; couple 44	We were together for 48 years. Her name was Carole. We met when we were relatively young. We spent a relatively uneventful life together, on the whole. (BH) Her mother had a child whom they named after me and who sadly died after birth. (TT)
T1	84%	1	**Relationship with healthcare teams**	13.54	soin [care] 211; traitement [treatment] 102; équipe [team] 78; rendez-vous [appointment] 38; loi [law] 32; soignant [caregiver] 32; psychologue [psychologist] 32; accompagner [to accompany] 31; remercier [to thank] 26; annoncer [to announce] 26	They knew her very well, so I went back to see the oncologist, the nurses, and the entire healthcare team. It was important for me to talk to the oncologist, too. (UH) I’d really like to go back and see the palliative care nurses there. I don’t know how to thank them because I think they took good care of her. (KE)
		2	**Absence and continuing presence in everyday life**	86.46	venir [to come] 8; envie [desire] 6; pareil [the same] 6; falloir [to have to] 5; arriver [to happen] 5; ensemble [together] 5; temps [time] 4; impression 4; endroit [place] 3; sortir [to go out] 3	I’m actually going through a difficult phase because it’s taking a long time and I miss her. (QN) Her presence is always there, even if we don’t necessarily talk about it. Even when we’re on the phone with friends, we mention her from time to time. (BH)
T2	74%	1	**Projects, family gatherings, and social life**	74.90	enfant [child] 21; aller [to go] 19; passer [to spend] 19; fille [daughter] 14; ami [friend] 13; faire [to do] 13; maison [house] 11; seul [alone] 11; ensemble [together] 10; famille [family] 9	People call me and say, “Hey, there’s a project, do you want to do it with us?” and I say yes. (NN) Those were the best years, the best year with my wife on vacation. (TT) It was Friday, […] my other son from Paris came for the weekend […], with her family, and we went to put flowers and all that again. (QB)
		2	**Palliative care and the circumstances of death**	14.11	soin [care] 251; douleur [pain] 99; souffrir [to suffer] 97; service [ward] 91; soignant [caregiver] 86; accompagner [to accompany] 78; souffrance [suffering] 74; équipe [team] 74; mourir [to die] 54; malade [patient] 48	I’ve been wanting to go back for a while now, both to thank people again. (TT) Yes, exactly, but I think here too we’re all different—some people withdraw a little into suffering and pain, and then they don’t dare talk about the illness. But they need to: all those people—the doctors, nurses, caregivers—they’re there to help us. (QN)
		3	**Reflections prompted by the research interview**	10.99	poser [to ask] 140; question 117; merci [thank you] 73; accord [agreement] 73; intéresser [to interest] 72; réponse [answer] 55; répondre [to answer] 40; réflexion 32; dialogue 24; injustice 24	We’ve exchanged our impressions quite a bit, and we’ve gotten answers to our questions—well, that’s my point of view. I hope everything we’re saying here will be useful to you. (QN)

Retention = percentage of elementary context units (ECUs) classified by the descending hierarchical classification for that wave; unclassified units are excluded from class-level percentages, which sum to 100% of classified ECUs within each wave. For each class, we report the ten forms with the highest *χ*^2^; when several forms share the same *χ*^2^ value, we list the first returned by the software

The thematic analysis was conducted on a dataset derived from this classification rather than on the full transcripts. For each class, the context units most strongly associated with it, as ranked and displayed by the software (up to 100 per class), were read in order of association. Following a codebook (coding reliability) approach [18, 19], the first two authors coded these segments independently, compared their coding, and developed themes through discussion, with a third researcher arbitrating where consensus was not reached.

The primary analysts were clinical psychologists with expertise in psycho-oncology, palliative care, and bereavement research. Their disciplinary background and clinical experience informed their sensitivity to the relational, emotional, and existential dimensions of grief and shaped their reading of which text segments were most relevant to each lexical class during coding. The analysis followed a critical realist epistemological framework, treating participants’ accounts as experiences that are both personally constructed and grounded in social and clinical realities. Because clinical background and prior familiarity with bereavement and palliative care could influence coding decisions in either direction, two safeguards consistent with a codebook (coding reliability) approach were used to manage this influence rather than treat it as an analytic resource: first, ALCESTE’s lexical classification, performed independently of any interpretive input, provided a corpus-driven starting point for the coding stage; second, independent coding by two analysts, systematic comparison of their coding, and arbitration by a third researcher were used to identify and resolve individual divergences in interpretation. Team discussions of these divergences made each analyst’s assumptions explicit and kept the resulting codes anchored to the classified material rather than individual preconceptions.

Due to the sensitive context of bereavement, member checking was not conducted to prevent additional distress.

Independent coding, consensus adjudication by a third researcher, and investigator triangulation supported analytical rigor.

The study adheres to the COREQ (Consolidated Criteria for Reporting Qualitative Research) [20] guidelines (see Supplementary File 1), ensuring transparency and methodological rigor. Combining ALCESTE’s statistical mapping of lexical patterns with codebook thematic coding offered objectivity in the classification stage, complemented by an interpretive reading of class content, to support a comprehensive understanding of participants’ experiences of grief [21]. Where lexical classification and thematic coding diverged—for instance when a lexical class spanned more than one substantive topic—resolution followed discussion between the two coders, with arbitration by the third researcher when needed.

## Results

The classification identified fifteen lexical classes across the six gender-by-wave runs. Retention ranged from 59 to 84% of elementary context units depending on the run. Table 3 reports the classes obtained for women and Table 4 those obtained for men, with class size expressed as a percentage of classified ECUs and the characteristic reduced forms with their *χ*^2^ values. Each subsection below corresponds to one numbered class.

### T0—illness trajectory and end-of-life experiences

#### Women

At T0, women’s narratives were structured around three main lexical classes, the largest of which (62.78% of classified ECUs) situated the illness within the couple’s and the family’s history rather than within the end-of-life period itself.

##### Class 1—couple and family history (62.78%)

Participants frequently situated the illness within a broader life narrative. They introduced the end-of-life experience through the couple’s and family’s history, using biographical details to make sense of the trajectory that led to the final moments.

“Both my husband and I were passionate about our work, and so that was a real asset. We shared this love of work and passed it on to our children”. (DC)

Women in this group often emphasized the time spent as a couple, passing on traditions to their children, and how their husband’s death affected their children.

##### Class 2—acute care and the final hours (23.68%)

A prominent class referred to emergency care, intensive care units, and the last hours spent at the patient’s bedside. These accounts included detailed descriptions of bodily signs (breathing patterns, medical devices, physical deterioration), emotional turmoil, and the urgency of decision-making.

“Between the noise of the machine and him, he was like this. I could see him suffocating. I ended up turning off the machine because with the machine I couldn’t think”. (DN)

Women often described acting under pressure, managing fear, and interpreting clinical signs while attempting to remain present and supportive. Their accounts were emotionally vivid, highlighting panic, helplessness, and the burden of witnessing physical decline.

##### Class 3—illness progression and treatments (13.54%)

Another lexical class concerned diagnostic stages, treatment lines (including experimental protocols), medical appointments, and fluctuating hopes. Women often described treatment cycles with precision—side effects, medical explanations, and therapeutic responses—while expressing alternating phases of expectation, hope, and disappointment.

“And so we are trying a new protocol again, which is not yet—it’s not quite an official protocol.” (MN)

The illness was portrayed as a complex, exhausting journey requiring continuous vigilance and coordination with multiple medical teams.

#### Men

Men’s narratives in T0 tended to emphasize a more factual, less emotionally embodied style.

##### Class 1—illness history and its clinical management (25.63%)

Men often provided structured accounts of the illness, emphasizing diagnoses, therapeutic options, and changes in medical condition. They described their supportive role (transportation and attendance at medical consultations) in a practical, task-oriented manner.

“So I was the one who took her there, and I also went to the appointments with the doctors, so we were both involved in terms of support. I was perfectly on top of every detail”. (HR).

The illness appeared as a sequence of steps to understand and manage rather than as an emotionally immersive experience.

##### Class 2—the last days and nights (52.48%)

The largest class in men’s T0 sub-corpus (52.48% of classified ECUs) also concerned the last days of life; men, however, described these moments with greater emotional distance. They mentioned regret, their presence in the previous days, and reassurance from healthcare professionals.

“She had been fighting for a pretty long time like that,[…] And since I had asked them not to call me because my children were asleep, and then, would it really change anything if I showed up at 2 a.m.?” (KN-E).

Descriptions were concise, with fewer sensory or corporeal details than in women.

##### Class 3—couple history and previous family losses (21.89%)

Several men also referred to long-standing relationships (often spanning several decades) and prior losses (parents, siblings).

“Yes, I was an only child because I lost my father at the age of… Yes, I’ve had tragedies in my life, too. I lost my brother when I was 7. He died when he was 10.” (BH)

### T1—early bereavement and emotional responses

#### Women

##### Class 1—everyday life with the children and the deceased’s belongings (23.40%)

In this sample, women frequently discussed managing children after the loss and the mental load associated with maintaining everyday life.

“All alone, I know I wouldn’t eat, for example, because I’m not hungry. I eat because I make food for my children. Making food for my children is a big deal.” (MN)

They described feeling “alone” and torn between their own emotional needs and the responsibility to appear strong for their children.

Memories, personal objects, photographs, and family rituals were central. Women often spoke of passing the deceased’s personal belongings to their children and of the children’s attachments to these items.

“He spent all his childhood vacations and adolescence there. […]. We took the children there several times when they were little, so I promised the children I would take them for a week.” (DN).

Family memory work emerged as a key aspect of early bereavement.

##### Class 2—making sense of the loss: unanswered questions and emotional experience (76.60%)

Participants expressed intense emotions: denial, fear, ambivalence, and difficulty accepting the death. Their narratives often returned to disbelief about the loss.

“But you’re still here, […] after all, but I realize that we can live as a family of three and that we can move forward without him, and that’s hard to accept.” (DN)

The emotional timeline appeared fragmented and unstable, characterized by recurring returns to the moment of death.

Women often revisited medical decisions (sedation, discontinuation of treatments, and artificial nutrition), expressing confusion or a need for clarity.

“If they hadn’t—it wasn’t me, by the way—if they hadn’t sedated him, he could have lived for quite a while, but in a lot of pain.” (MC)

These reflections were sometimes linked to traumatic images that remained vivid after the death.

#### Men

##### Class 1—relationship with healthcare teams (13.54%)

Several men discussed follow-up appointments, administrative steps, and offers of psychological support.

“We talked a little bit—it was when the head doctor of the palliative care department held a teleconference with the children. It was about a week before she passed away, and well.”(QB).

This reflected an orientation toward institutional and procedural aspects of the illness.

##### Class 2—absence and continuing presence in everyday life (86.46%)

Many men expressed a persistent sense of absence grounded in everyday habits and shared routines. This longing focused more on the life lived together than on the final moments.

“Even though we had experienced a lot together, we still felt like we wanted to experience more. We still felt this lack of, I don’t know, closeness in our life together.” (UH)

Men more often framed bereavement as a reorganization of daily life rather than an emotional struggle focused on the circumstances of death.

### T2—reorganization of daily life and future perspectives

#### Women

##### Class 1—evaluation of care and hospital services (19.83%)

Many women expressed strong appreciation for palliative care teams, describing this setting as humane and gentle, in contrast to the stressful atmosphere of emergency units.

“There’s nothing to complain about. I have nothing to complain about regarding palliative care.” (QC)

They emphasized the quality of support provided at the time of death.

##### Class 2—life reorganization and the question of the future (80.17%)

Women tended to express hesitation regarding the possibility of future relationships, due to their emotional attachment to the deceased, the presence of children, and the difficulty of imagining another bond.

“He told me he doesn’t want to get attached to them, because he’s nothing to them and will never be anything to them. I told him: today you’re nothing, true, on paper you’re nothing. But in practice, you’re not nothing — because you’re part of my children’s daily life. So in practice, you do have a status. (MN).

Future planning was approached with caution, often constrained by continuing emotional loyalty to their partner.

#### Men

##### Class 1—projects, family gatherings and social life (74.90%)

In this sample, men discussed numerous projects; many re-engaged actively with social environments, reflecting an action-oriented adjustment style.

“In July, we’re going to go on another motorcycle trip, but this time with a different group of friends. We’re going to the Mountains for four or five days.”(TT)

##### Class 2—palliative care and the circumstances of death (14.11%)

Several men described their understanding of palliative sedation, anticipatory directives, and the distinction between the death itself and the medical process leading to it.

“When you consider the quality of care, the stay, etc., the attention of the caregivers who carry and observe how the patients are treated, in addition to sedation, will ensure there is no suffering. Well, that’s still something positive for… Well… It’s positive for the spouse or family, at least for me; it was positive.”(PA).

This framing allowed them to contain guilt and emphasize that the purpose of sedation was relief from suffering.

##### Class 3—reflections prompted by the research interview (10.99%)

Some men adopted a more generalized perspective, reflecting on the experiences of other bereaved individuals and on the role of palliative care in alleviating suffering; the interview appeared to function as an occasion for this comparative, meaning-making reflection rather than being its object.

“We’ve exchanged our impressions quite a bit, and we’ve gotten answers to our questions—well, that’s my point of view. I hope everything we’re saying here will be useful to you.” (QN).

## Discussion

The longitudinal analysis of spouses’ narratives highlights the evolving and context-dependent nature of grief following cancer, with gender emerging as a meaningful lens rather than a fixed determinant. The final days and the circumstances of the death were central to the narratives of both men and women at T0 and were prominent among the lexical classes of both sub-corpora (Tables 3 and 4). What distinguished the two groups was therefore not the topic but the register in which it was recounted: women returned to images of bodily deterioration and to the sensory detail of care in emotionally rich and embodied terms, whereas men recounted the same events at a greater emotional distance and in a largely instrumental idiom. Women also more often situated the illness within the couple’s and the family’s biography, which formed the most prominent class in their narratives at this wave. These expressions resonate with literature showing that women often engage more in emotional processing and relational meaning-making, especially when deeply involved in caregiving [22, 23]. Yet our findings suggest that these narratives do not simply reflect emotionality; they also articulate the cognitive load of decision-making, exposure to medicalized suffering, and the responsibility of maintaining family continuity. Within this sample, men more often narrated bereavement through pragmatic or instrumental frames—emphasizing actions, medical information, or disruptions to routines—consistent with existing descriptions of instrumental coping [24]. Nevertheless, their accounts also included moments of emotional rupture and existential insecurity, supporting recent calls to consider gender differences as fluid, overlapping, and shaped by situational demands rather than binary models [25]. At T2, for instance, one woman’s narrative (OS) was instead anchored in the factual, clinical register more typical of men’s accounts in this sample, centering on hospital services and care coordination rather than emotional processing; conversely, one man’s narrative (HR) was closely aligned with the register more typical of women, centering on suffering, accompaniment, and the circumstances of the death itself. These within-gender departures from the dominant register illustrate that the patterns described above reflect group-level tendencies rather than fixed categories and support a cautious reading of the “rigid binary interpretation” caveat noted above.

Before interpreting these patterns further, several alternative explanations for the observed contrast deserve consideration alongside the gendered-narrative-registers hypothesis. The same two female interviewers conducted all interviews using an identical guide, and interviewer turns were excluded from the corpus submitted for classification, so the contrast cannot be attributed to interviewer gender or question wording. A same-gender dyad effect nonetheless remains possible, since female participants were interviewed by same-gender interviewers while male participants were not. Interviews with women were also longer at every wave (mean 83.2, 60.6, and 55.4 min at T0, T1, and T2, respectively, vs. 63.4, 43.9, and 41.7 min for men), and the resulting sub-corpora submitted to classification differed appreciably in size (984 vs. 682 Ko at T0, 550 vs. 270 Ko at T1, and 530 vs. 428 Ko at T2, for women and men respectively), although women were fewer (10 vs. 12); retention varied from 59 to 84% across runs (Supplementary Table S1). Because descending hierarchical classification is sensitive to corpus size, the number and granularity of classes are not directly comparable across gender, and this asymmetry—rather than gender per se—could in principle account for part of the contrast in register. For these reasons, we describe our findings as gendered narrative registers observed within this sample and in this interview context rather than gendered grief pathways, and we regard differences in expressive register as compatible with, but not evidence of, differences in underlying grief processes.

Across genders, the end-of-life trajectory had a decisive influence on later grief. Participants frequently linked their distress to emergency settings, rapid deterioration, or uncertainty surrounding palliative sedation. Consistent with growing evidence, the clarity of communication from healthcare teams shaped how spouses retrospectively interpreted these events [26]. When sedation was well explained, spouses tended to integrate it as a compassionate intervention; when communication was insufficient, they reported unresolved questions, guilt, or intrusive recollections. This pattern echoes findings that end-of-life decision-making significantly affects grief adjustment and may contribute to prolonged grief symptoms when poorly understood [27].

The longitudinal design also illuminates the temporal evolution of grieving processes. Early bereavement was often marked by emotional disorientation and intrusive memories—especially among women—whereas later phases showed a gradual shift toward reorganization of daily life and future planning, more evident among men. These trajectories align with longitudinal studies indicating that acute grief typically gives way to adaptive functioning over time, even when sorrow persists [13]. However, for spouses exposed to traumatic or confusing end-of-life circumstances, grief appeared more complex and slower to integrate, suggesting that anticipatory grief and medicalized dying can complicate adjustment.

A further dimension concerns the economic consequences of widowhood. Women in this sample reported far more substantial financial losses than men, reflecting structural gender inequalities documented in population studies [3, 4]. These disparities underscore that bereavement unfolds not only in emotional and relational domains but also within socioeconomic constraints that may intensify vulnerability.

Together, these findings support the need for bereavement care that is sensitive to gendered patterns while avoiding stereotyped assumptions. Clear, early communication during the end-of-life phase—particularly about sedation and clinical expectations—appears crucial for later adjustment. Tailored support may be especially important for individuals confronted with traumatic images, ambiguous medical decisions, or high caregiving burden. Meaning-centered and narrative approaches may help address the intersection of emotional, embodied, and cognitive dimensions that characterize spousal grief in this context.

At the same time, several limitations should be noted. The qualitative design and small sample limit generalizability and interpret findings in terms of transferability to comparable contexts rather than statistical generalizability. The analysis was restricted to the twenty-two participants with a complete three-wave dataset available at the time of analysis. Although included and nonincluded participants were comparable on the baseline characteristics available, these data were incomplete for the nonincluded group, and the comparison therefore rests on a subset of them; inclusion also depended on recruitment date and on the progress of manual transcription, so the included participants were among those recruited earliest, and we cannot rule out that they differ from later recruits in ways related to the recruitment period. We operationalized gender in binary terms (men/women), which may not capture the diversity of participants’ experiences. The possible same-gender interviewer dyad effect discussed above remains unverified, as we did not systematically analyze probing style. Unbalanced sub-corpus sizes (12 men vs. 10 women, and 10–12 interviews per gender per wave) may also have affected the sensitivity and stability of the ALCESTE lexical classification. Furthermore, the thematic analysis was conducted on the context units most strongly associated with each lexical class, taken in order of association, rather than on the full transcripts; these segments are by construction the most typical of their class, and material weakly associated with a class, along with ECUs not classified by the algorithm, did not enter the thematic dataset. The themes therefore describe the most densely characteristic material of the corpus rather than its full range and may present the classes as more internally homogeneous than the underlying interviews. Verbatim quotes were translated from French for publication; nuances of the original wording may not be fully captured. Variability in clinical pathways and sedation practices further complicates interpretation of the findings.

Future research should use larger, more diverse samples, integrate mixed-methods designs, and examine how relational history, caregiving roles, and end-of-life communication jointly shape bereavement trajectories. Developing more flexible theoretical models that combine gender with emotional, relational, clinical, and structural factors may support a more comprehensive understanding of spousal grief.

## Conclusion

Within this sample and interview context, spousal bereavement in the oncological and palliative setting was associated with gendered narrative registers alongside caregiving roles and the quality of end-of-life communication. While women and men often relied on different expressive registers, these patterns were fluid and context-dependent rather than fixed. Clear information about clinical decisions—particularly palliative sedation—plays a pivotal role in how spouses make meaning of the death. Supporting bereaved individuals requires an individualized, gender-sensitive approach that acknowledges emotional, relational, and structural vulnerabilities and the profound impact of medicalized dying on the grieving process.

## Supplementary Information

Below is the link to the electronic supplementary material.

ESM 1 (DOCX 12.1 KB)

ESM 2 (DOCX 26.7 KB)

## Data Availability

The datasets used and/or analyzed during the current study are available from the corresponding author upon reasonable request.
