# Supplementary material for: End-of-life sedation and gendered narrative registers: a longitudinal qualitative study of spouses bereaved by cancer
Source: Support Care Cancer. 2026 Sep 17;34(10):980. doi: 10.1007/s00520-026-11224-6 (PMC13582097; doi:10.1007/s00520-026-11224-6)
Supplement: Supplementary file 2 [file 520_2026_11224_MOESM2_ESM.docx]

**Supplementary Table S1.** ALCESTE classification parameters and retention by run.

| **Wave** | **Gender** | **N participants** | **Corpus size** | **Stable classes** | **Retention (% classified ECUs)** |
| --- | --- | --- | --- | --- | --- |
| T0 | Men | 12 | 682 Ko | 3 | 67% |
| T0 | Women | 10 | 984 Ko | 3 | 83% |
| T1 | Men | 12 | 270 Ko | 2 | 84% |
| T1 | Women | 10 | 550 Ko | 2 | 59% |
| T2 | Men | 12 | 428 Ko | 3 | 74% |
| T2 | Women | 10 | 530 Ko | 2 | 81% |

*Note. ECU = elementary context unit. Corpus size is reported as generated by ALCESTE (software file size in kilobytes). Retention refers to the percentage of ECUs classified into stable lexical classes for each gender-by-wave sub-corpus.*
